# Supplementary material for: Improving rechargeable magnesium batteries through dual cation co-intercalation strategy
Source: Nat Commun. 2024 Jan 12;15:492. doi: 10.1038/s41467-023-44495-2 (PMC10786895; doi:10.1038/s41467-023-44495-2)
Supplement: Supplementary file 1 — Supplementary Information [file 41467_2023_44495_MOESM1_ESM.pdf]

Supplementary Information

Improving Rechargeable Magnesium Batteries through Dual Cation Co-Intercalation Strategy

Ananyo Roy [1], Mohsen Sotoudeh [2], Sirshendu Dinda [1], Yushu Tang [3,4], Christian Kübel [1,3,4], Axel Groß [1,2], Zhirong Zhao-Karger[1,3], Maximilian Fichtner [1,3], Zhenyou Li [1,5,6,7] *

[1] Helmholtz Institute Ulm (HIU), Helmholtzstraße 11, 89081 Ulm, Germany

[2] Institute of Theoretical Chemistry, Universität Ulm, Oberberghof 7, 89081 Ulm, Germany

[3] Institute of Nanotechnology (INT), Karlsruhe Institute of Technology (KIT), Hermann-von-Helmholtz-Platz 1, 76344 Eggenstein-Leopoldshafen, Germany

[4] Karlsruhe Nano Micro Facility (KNMF), Karlsruhe Institute of Technology (KIT), Eggenstein-Leopoldshafen, Germany

[5] Qingdao Institute of Bioenergy and Bioprocess Technology, Chinese Academy of Sciences, No. 189 Songling Road, Laoshan District, Qingdao, Shandong 266101, China

[6]Shan-dong Energy Institute, Qingdao 266101, China

[7]Qingdao New Energy Shandong Laboratory, Qingdao 266101, China

*Correspondence to: zhenyou.li@kit.edu

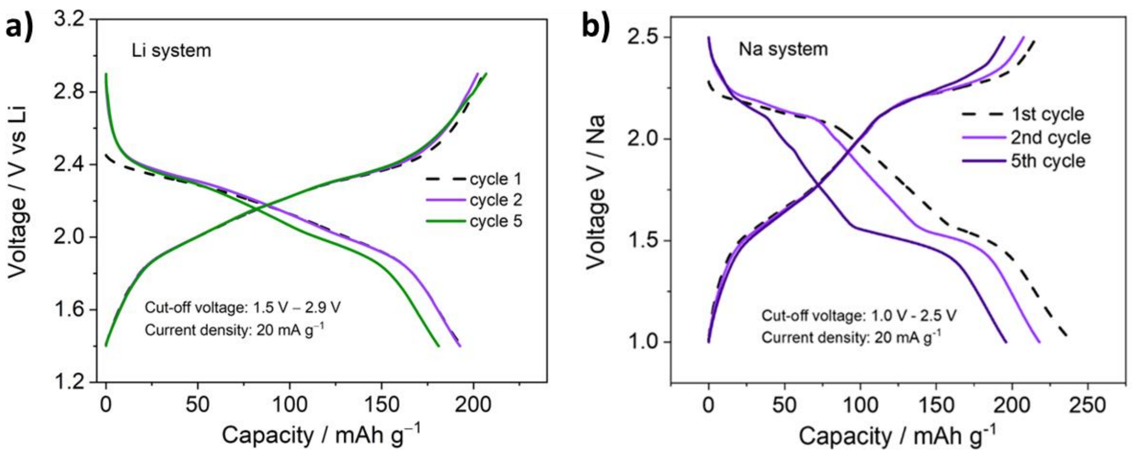

Supplementary Figure 1: Electrochemical voltage profile of TiS_2. a) Li system, configured by implementing Li metal anode and Li[B(hfip)$_4$] electrolyte. **b)** Na system, configured by implementing Na[B(hfip)$_4$] electrolyte in a Na half-cell setup.

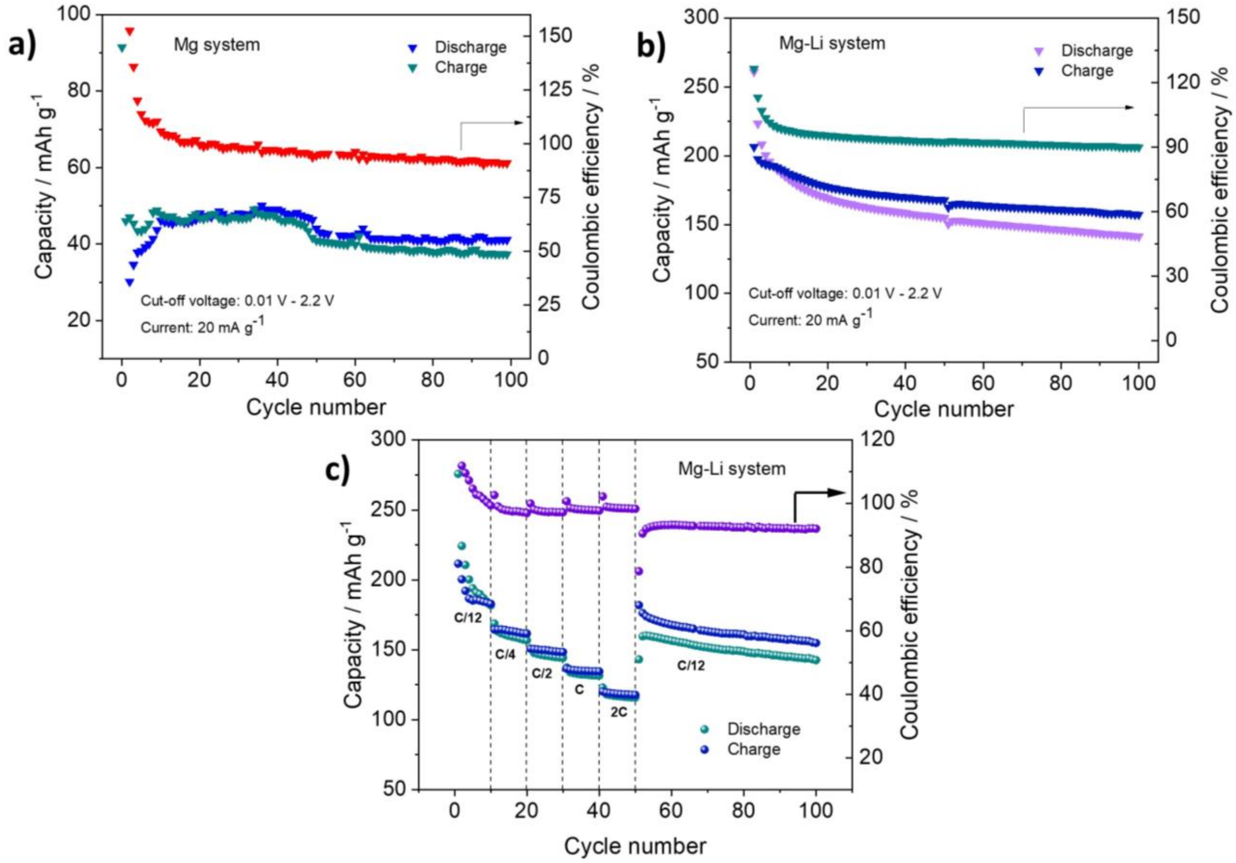

Supplementary Figure 2: Cycling performance and coulombic efficiency of TiS_2. a) Mg system, configured by incorporating Mg metal anode and $Mg[B(hfip)_4]_2$ electrolyte. **b**) Mg-Li system, configured by incorporating Mg metal anode and $Mg[B(hfip)_4]_2$ – $Li[B(hfip)_4]$ dual salt electrolyte. **c)** Rate test of TiS_2 in the Mg-Li system.

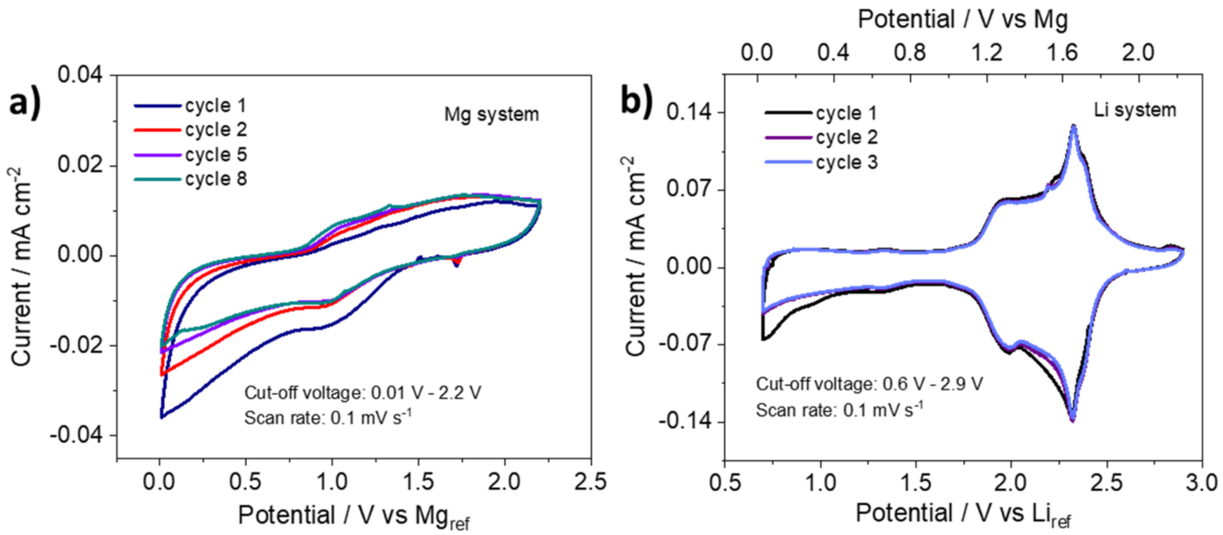

Supplementary Figure 3: Three-electrode cyclic voltammogram of TiS_2. a) Mg system with Mg reference electrode (Mg_{ref}). **b)** Li system with Li reference electrode (Li_{ref}).

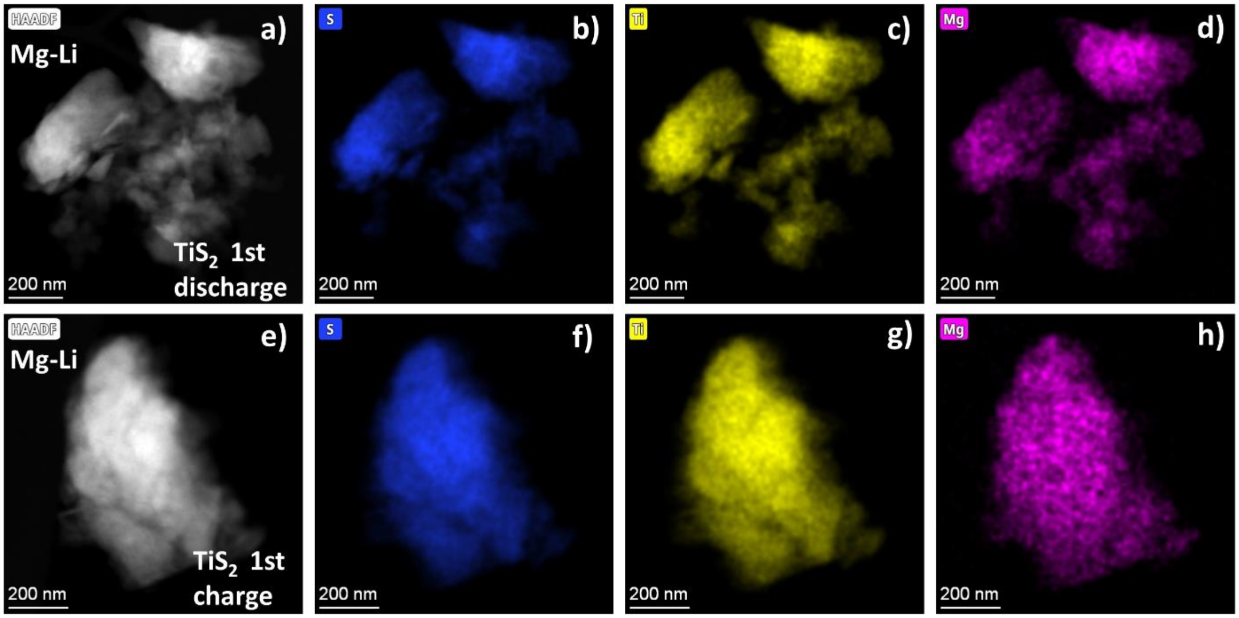

Supplementary Figure 4: STEM-HAADF and EDX analysis of cycled TiS_2 in the Mg-Li system. a) 1st discharge. **e)** 1st charge. Elemental mapping showing the distribution of S (blue), Ti (yellow) and Mg (pink) **(b-d)** after 1st discharge and **(f-h)** 1st charge.

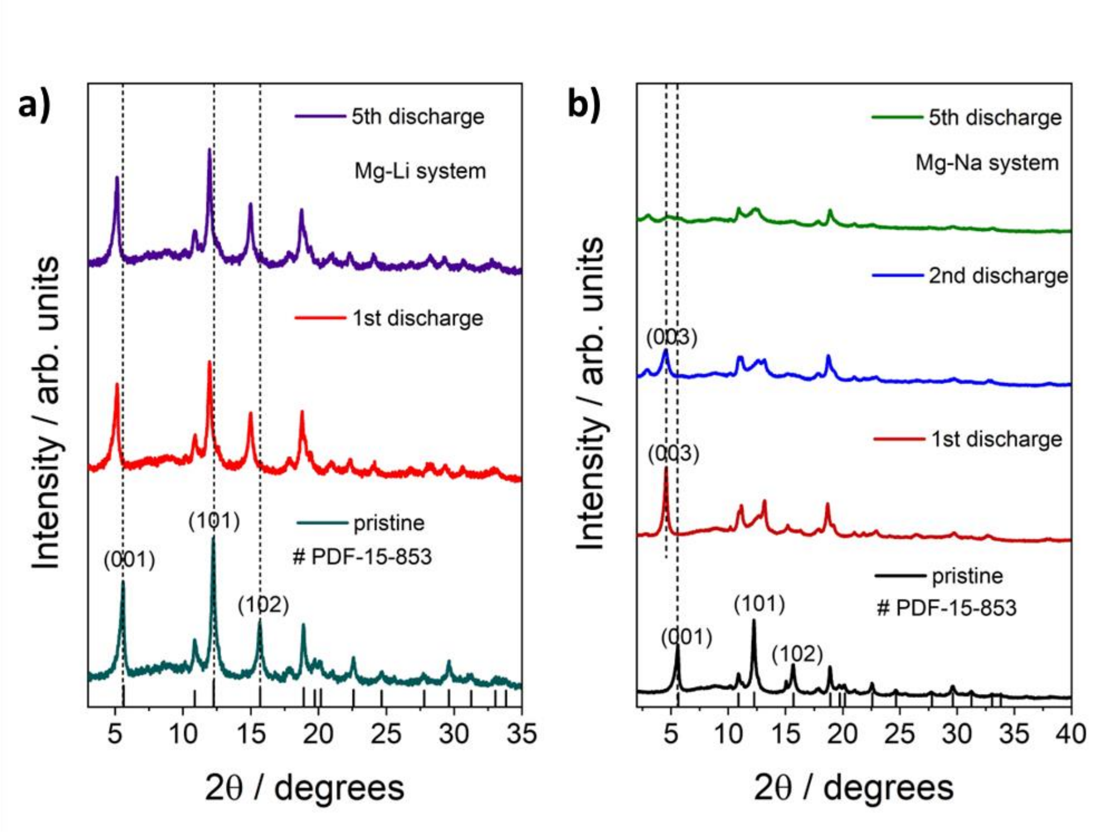

Supplementary Figure 5: Post mortem XRD of TiS_2. a) Mg-Li system after 1st and 5th discharge. **b)** Mg-Na system after 1st 2nd and 5th discharge.

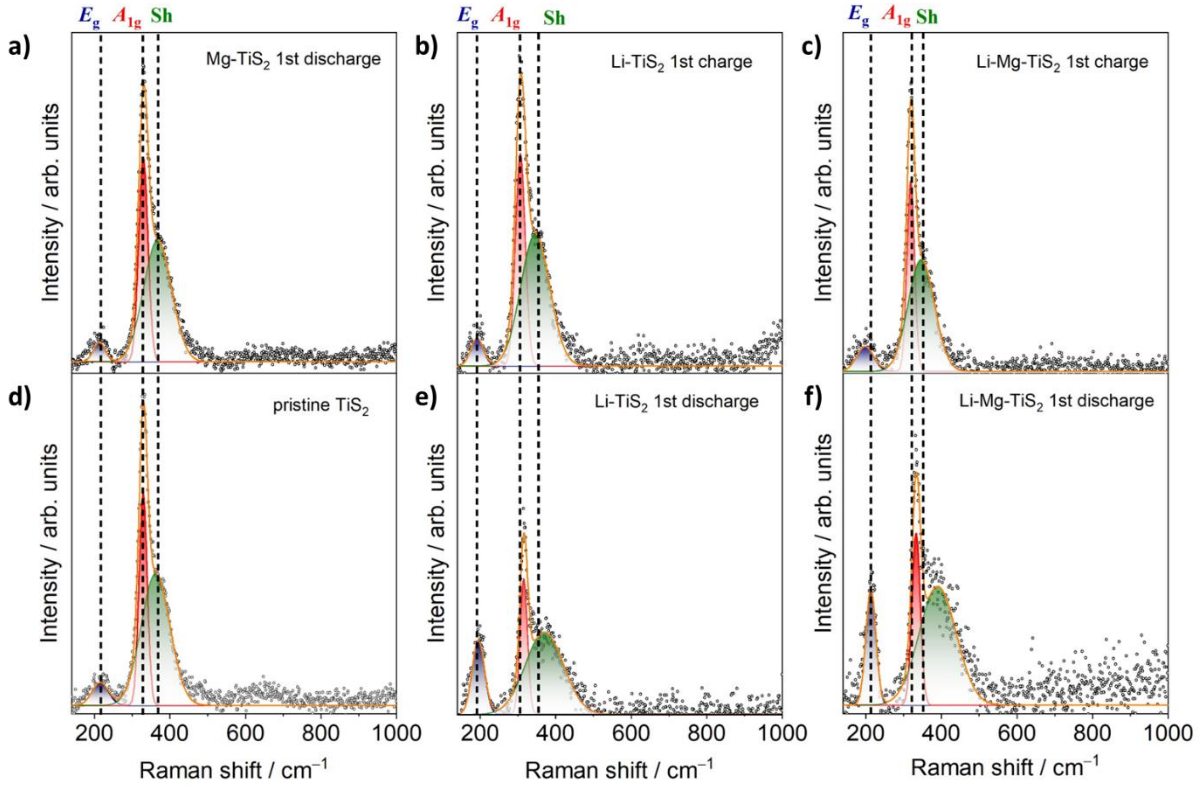

Supplementary Figure 6: Fitted Raman spectra of TiS_2. Comparing the Raman active in-plane E_{1g} and the out-of-plane A_{1g} and Sh mode frequencies in **d)** pristine sample, **a)** Mg system after 1st discharge; Li system after **e)**1st discharge and **b)** 1st charge; Mg-Li system after **f)** 1st discharge and **c)** 1st charge.

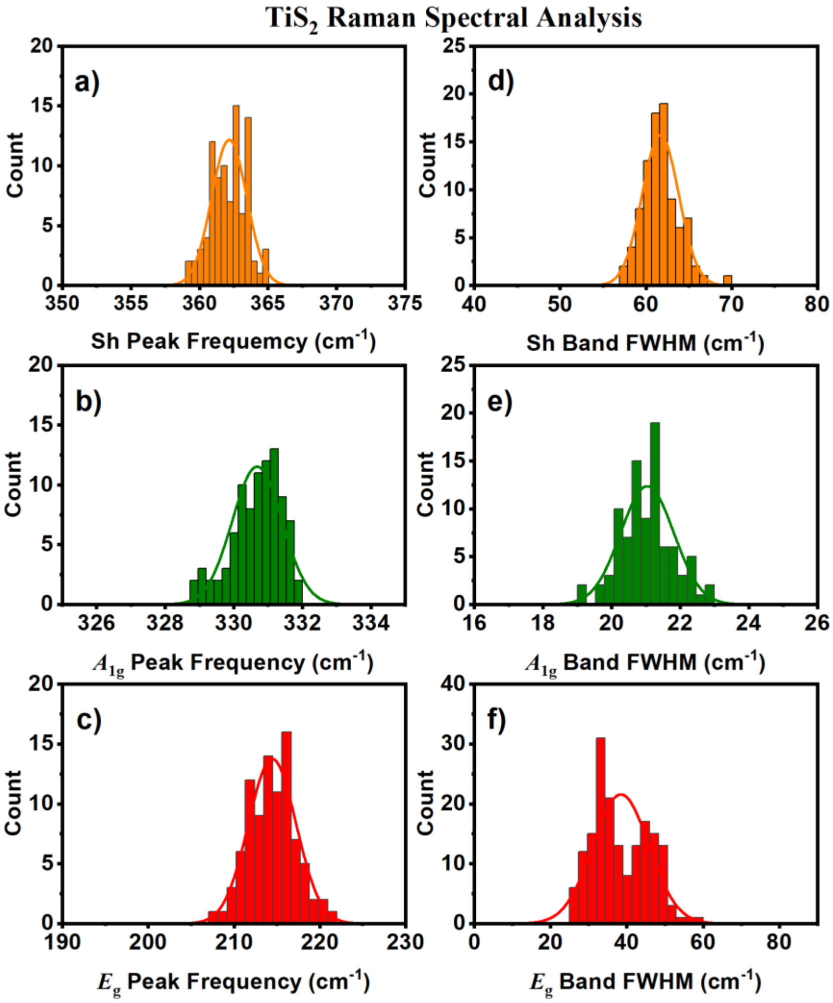

Supplementary Figure 7: Statistical evaluation and validation of the collected Raman spectra. Peak position of **a)** Sh mode, **b)** A_{1g} mode and **c)** E_g mode. FWHM of **d)** Sh band, **e)** A_{1g} band and **f)** E_g band.

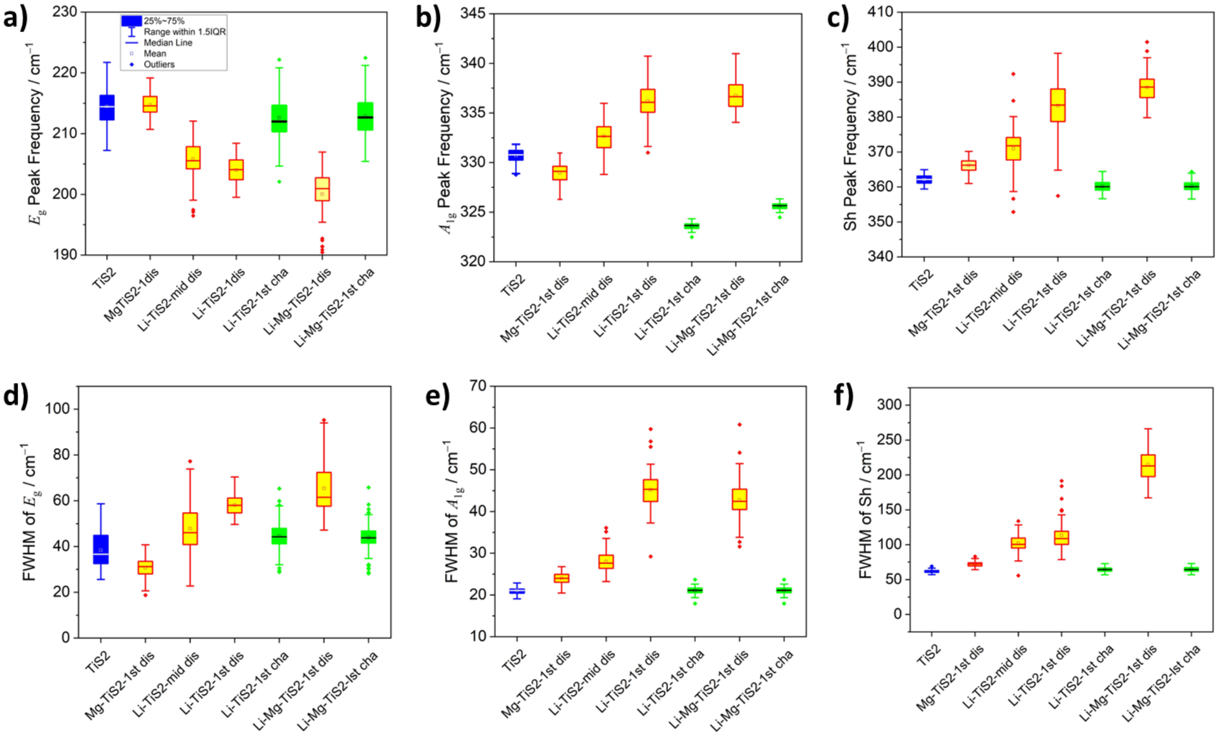

Supplementary Figure 8: Peak frequency and FWHM distribution using box chart showing median, standard deviation and outliers. Pristine TiS_2 (blue), discharged TiS_2 (yellow) and charged TiS_2 (green) samples showing **a, c)** E_g mode, **b, e)** A_{1g} mode and **c, d)** Sh mode.

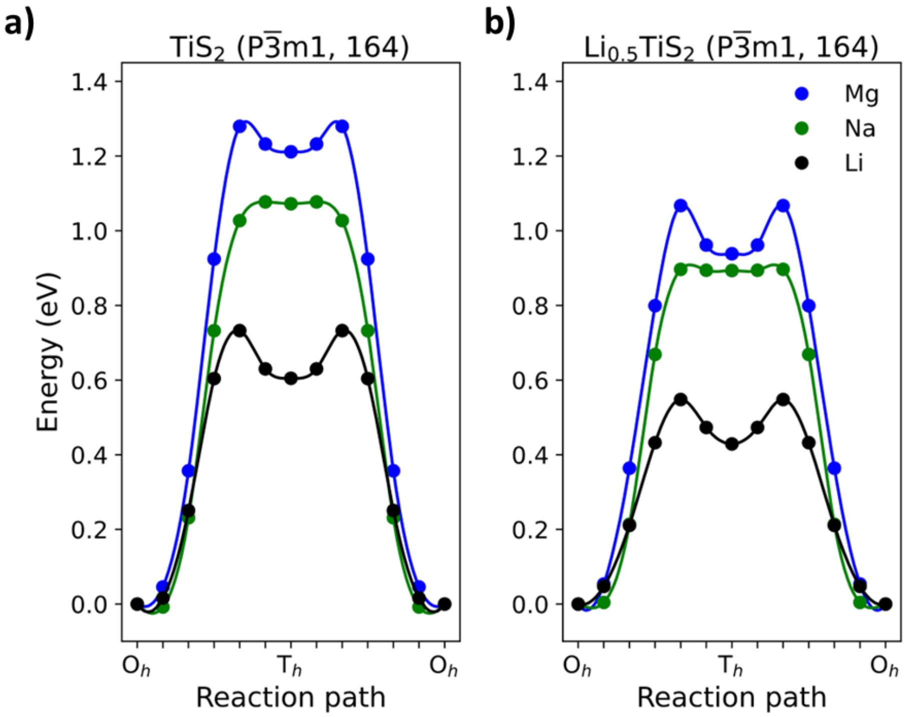

Supplementary Figure 9: Calculated activation barriers. Mg, Na and Li ion diffusion in bulk **a)** O3-TiS_2 and **b)** O3-$Li_{0.5}TiS_2$.

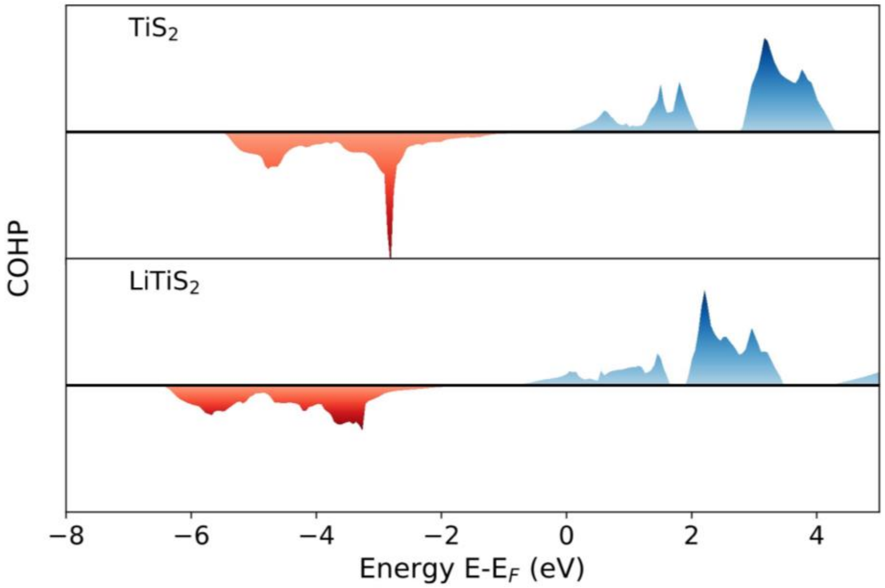

Supplementary Figure 10: The Crystal Orbital Hamiltonian (COHP) analysis between a Ti-d orbital and S-p state. COHP values manifest as both negative (indicating bonding, shown in red) and positive (indicating anti-bonding, shown in blue) interactions, positioned below and above the horizontal black line, respectively. The zero energy is aligned with the valence band top.

Supplementary Table 1: Stoichiometry of TiS_2 electrodes normalized by Ti measured by ICP-OES after 1st discharge, 1st charge, 5th discharge and 5th charge of the Mg-Li and single ion Mg systems.

	**Stoichiometry**	
**Samples**	**Mg-Li cell**	**Mg cell**
1st discharge	$Mg_{0.24}\ Li_{0.35}\ TiS_{2.04}$	$Mg_{0.16}\ TiS_{1.78}$
1st charge	$Mg_{0.17}\ Li_{0.01}\ TiS_{2.02}$	$Mg_{0.11}\ TiS_{1.83}$
5th discharge	$Mg_{0.40}\ Li_{0.53}\ TiS_{2.26}$	$Mg_{0.27}\ TiS_{2.01}$
5th charge	$Mg_{0.27}\ Li_{0.007}\ TiS_2$	$Mg_{0.19}\ TiS_{2.07}$

***S*upplementary Table 2:** Stoichiometry of TiS_2 electrodes, normalized by Ti, that were measured by ICP-OES at different states of charge of the Mg-Na system.

**Samples**	Discharge 1.13 V	Discharge 0.6 V	Discharge 0.01V	Charge 2.2 V
**Stoichiometry**	$Mg_{0.11}$ $Na_{0.46}$ $TiS_{1.97}$	$Mg_{0.13}$ $Na_{0.75}$ $TiS_{2.05}$	$Mg_{0.13}$ $Na_{0.81}$ $TiS_{1.86}$	$Mg_{0.125}$ $Na_{0.09}$ $TiS_{1.98}$

Supplementary Table 3: The calculated lattice constants (a, b, c) as well as Ti-S, Ti-Ti, A-S, and A-A bond lengths in angstroms for the considered layered compounds. A denotes intercalated atoms (Li, Mg, and Na). The angle between the anions has been represented by $S - \widehat{S} - S$ in the unit of degree. The corresponding space group of the considered compounds are also listed.

Compound	a (Å)	b (Å)	c (Å)	Ti-S (Å)	Ti-Ti (Å)	A-S (Å)	A-A (Å)	$S - \widehat{S} - S$	Space group
**TiS_2**	3.394	3.394	5.488	2.413	3.394/5.488	-	-	180.00°	P$\bar{3}$m1
**$LiTiS_2$**	3.408	3.408	6.013	2.451	3.408/6.013	2.502	3.408	180.00°	P$\bar{3}$m1
**$MgTiS_2$**	3.458	3.458	6.106	2.495	3.458/6.106	2.531	3.458	180.00°	P$\bar{3}$m1
**$Mg_{0.5}TiS_2$**	3.408	6.718	18.860	2.413/2.438	3.414/6.572	2.580	3.408	176.85°	R3m
**$Mg_{0.5}TiS_2$**	3.395	3.395	17.809	2.418/2.446	3.395/6.252	2.450	6.791	178.64°	R$\bar{3}$m
**$NaTiS_2$**	3.527	3.527	19.584	2.474	3.527/6.838	2.758	3.527	180.00°	R$\bar{3}$m
**$Na_{0.5}TiS_2$**	3.387	3.387	19.728	2.407	3.387/6.861	2.711	6.774	180.00°	R3m

Supplementary Table 4: The calculated charges using PBE functional.

Element	Charge (VASP) TiS_2	Charge (Bader) TiS_2	Charge (VASP) $LiTiS_2$	Charge (Bader) $LiTiS_2$
**Li**	-	-	2.074	2.129
**Ti**	8.574	8.291	8.585	8.427
**S**	3.745	6.854	3.795	7.221
**S**	3.745	6.855	3.795	7.221

Supplementary Table 5: The calculated charges using HSE06 functional.

Element	Charge (VASP) TiS_2	Charge (Bader) TiS_2	Charge (VASP) $LiTiS_2$	Charge (Bader) $LiTiS_2$
**Li**	-	-	2.067	2.116
**Ti**	8.378	7.997	8.506	8.230
**S**	3.804	7.002	3.844	7.326
**S**	3.804	7.001	3.844	7.327
